# Mindfulness and Metta-based Trauma Therapy (MMTT): Initial Development and Proof-of-Concept of an Internet Resource

**DOI:** 10.1007/s12671-015-0402-y

**Published:** 2015-04-09

**Authors:** Paul Frewen, Nicholas Rogers, Les Flodrowski, Ruth Lanius

**Affiliations:** 1 Department of Psychiatry, University Hospital, University of Western Ontario, 339 Windermere Rd (Room B3-264), London, ON N6A 5A5 Canada; 2 Department of Psychology, University Hospital, University of Western Ontario, London, ON N6A 5A5 Canada

**Keywords:** Mindfulness, Metta (lovingkindness), Meditation, Internet therapy, Posttraumatic stress disorder (PTSD), Depression, Anxiety, Dissociation

## Abstract

Trauma and stressor-related disorders, including post-traumatic stress disorder (PTSD) and related comorbid disorders such as anxiety, depression, and dissociative disorders, are difficult to treat. Mindfulness-based clinical interventions have proven efficacy for mental health treatment in face-to-face individual and group modalities, although the feasibility and efficacy of delivering these interventions via the internet has not been evaluated. The present research developed mindfulness and metta-based trauma therapy (MMTT) as an internet resource to support the practice of mindfulness and metta (lovingkindness) meditations for self-regulation and healing from trauma and stressor-related disorders. In the present “proof-of-concept” study, research participants (*n* = 177) recruited online practiced mindfulness and metta meditations and related therapeutic exercises available via the website and rated their perceived credibility as interventions for improving self-regulation and well-being and reducing PTSD symptoms, anxiety, depressive, and dissociative experiences, as well as their experienced ease, helpfulness, and informational value. Results suggest that, independent of level of self-reported current and past psychiatric history and PTSD symptoms, participants considered the MMTT website as a credible and helpful therapeutic intervention for improving self-regulation and well-being and reducing PTSD, anxiety, depression, and dissociation. Overall, participants considered guided and non-guided meditation practices more helpful than a journaling exercise, and participants with increased PTSD symptoms preferred metta (lovingkindness) meditations less than other participants. We conclude that MMTT should be piloted in clinical trials as an adjunctive intervention to evidence-based treatments for persons with mood, anxiety, and trauma and stressor-related disorders, as well as more generally as an online resource to support self-regulation and well-being practices.

## Introduction

Buddhist psychology recognizes suffering as a universal human experience, and modern clinical psychology research tends to agree that the majority of people worldwide are likely to encounter one or more traumatic events and/or relationships within their lifetime. Prevalence rates for trauma exposure approximate 67 % internationally (Karam et al. 2014; McLaughlin et al. 2014; Norris and Sloan 2014; Stein et al. 2014), and epidemiological studies suggest that rates in North America may be even higher (e.g., Canada, 76 %; Van Ameringen et al. 2008; United States, 90 %; Kilpatrick et al. 2013). Common traumatic stressors include motor vehicle accidents, childhood maltreatment, intimate partner violence, being physically or sexually assaulted, witnessing death or serious injury, or experiencing the unexpected death of a loved one. Notwithstanding the pervasiveness of psychological trauma in human life, some persons clearly suffer more greatly than others, with many ultimately developing post-traumatic stress disorder (PTSD) and related comorbid disorders, including anxiety disorders, depression, and dissociative disorders. Current evidence-based psychological treatments for trauma and stressor-related disorders are primarily cognitive behavioral in nature; however, even gold-standard treatments for PTSD often fall short, with reviews suggesting that approximately one in every two-to-three people with PTSD who receive evidence-based treatments will fail to show clinically significant improvement (e.g., Bradley et al. 2005). Moreover, drop-out rates for PTSD treatment are woefully high, approximating one in every four-to-five participants (and can be even higher, e.g., 38 % in Schnurr et al. 2007; reviews by Hembree et al. 2003; Schottenbauer et al. 2008). Researchers are therefore also evaluating non-traditional treatment modalities for trauma and stressor-related disorders including mindfulness-based interventions and internet-based treatment.

Clinicians and researchers have described a number of ways by which mindfulness-based therapies may exert therapeutic benefits for people with PTSD (Lang et al. 2012; Thompson et al. 2011). These include (1) improving attention and concentration; (2) improving participants’ abilities to focus on the present and away from traumatic memories and other sources of rumination and anxiety; (3) altering of cognitive style (e.g., becoming less judgmental and more compassionate toward oneself and others); (4) lowering psychophysiological arousal; and (5) lowering of anhedonia and emotional numbing. A similar set of potential therapeutic benefits of mindfulness-based therapies for persons with trauma-related dissociative disorders was articulated by Zerubavel and Messman-Moore (2015). However, despite a sound treatment rationale, relatively few studies have examined the efficacy of mindfulness-based treatments for PTSD to date. Moreover, most published studies are limited by small sample sizes, inclusion of traumatized persons both with and without PTSD, and only pre-post (non-randomized) or wait-list control (rather than active control) designs. Acknowledging these limitations, research accumulated to date generally provides support for mindfulness-based stress reduction (MBSR, Kabat-Zinn 1990) and mindfulness-based cognitive therapy (MBCT, Segal et al. 2002) as interventions for persons with PTSD or PTSD-related symptoms either in combat veterans (Kearney et al. 2012; King et al. 2013; Niles et al. 2012) or women with histories of childhood abuse (Goldsmith et al. 2014; Kimbrough et al. 2010) or intimate partner violence (Bermudez et al. 2013; Dutton et al. 2013).

Complementing mindfulness-based interventions, *metta* meditation practices involve intentionally developing kindness and compassion as directed toward both oneself and others through the verbal rehearsal, visualization, and embodiment of positive intentions and aspirations (e.g., for freedom from enmity, mental and physical suffering, and for good will, joy, and peace). Such practices may also be beneficial to PTSD treatment toward the aim of directly reducing trauma-related dysphoric experiences including shame, anhedonia, and emotional numbing, as well as for increasing resilience (reviews by Hinton et al. 2013; Hofmann et al. 2011; Thompson et al. 2011). In support, Kearney et al. (2013) found that a 12-week course in metta meditation, conducted as an adjunct to treatment as usual, reduced PTSD symptoms and increased self-compassion and mindfulness traits in combat veterans.

In addition to examining mindfulness- and metta-based therapies for PTSD, researchers are examining the efficacy of non-traditional modalities for provision of treatment including internet-based treatment. An advantage of internet therapy is that it requires few clinical resources, with some evaluated programs being entirely automated and others entailing at most only a small number of face-to-face, telephone, or email communications per participant. Moreover, the ability for clinician-to-participant dyadic communication to be completed, if at all, solely through indirect methods can eliminate certain barriers that may otherwise prevent or discourage persons from receiving treatment where indicated, for example, due to social stigma associated with receiving mental health care, time and place accessibility, the financial cost of treatment, and the paucity of appropriately trained mental health professionals to deliver evidence-based treatment (see Boasso et al. 2014, for review). To date, psychological treatments for trauma and stressor-related disorders delivered via the internet generally involve exercises derived from cognitive behavior therapy (CBT), including narrative exposure therapy (structured writing about one’s traumatic experiences) and certain forms of relaxation training.

Previous studies have found internet service delivery of CBT for PTSD to be both highly acceptable and effective. In fact, research shows that PTSD outcomes for online treatment are often of a comparable magnitude to those found for the same kinds of interventions when delivered face to face, as has been generally found for internet therapy of other affective disorders (Andersson and Cuijpers 2009; Cowpertwait and Clarke 2013; Cuijpers et al. 2011; Spek et al. 2007). Indeed, the first study of internet treatment of PTSD was completed more than a decade ago and demonstrated, among 20 college students previously exposed to interpersonal violence, that providing CBT interventions for emotion regulation in addition to exposure-based narrative writing about traumatic events conducted via email resulted in clinically significant reductions in post-treatment PTSD symptoms (Lange et al. 2000). Since that time, research has suggested that individuals are indeed interested to receive internet-based treatments of PTSD (e.g., Spence et. al., 2011), and a number of additional studies have provided general support for internet CBT interventions for PTSD, although most studies have only compared outcomes via pre-post or non-active control designs (e.g., with supportive counseling; see Boasso et al. 2014, for review). However, we are aware of no internet-based mindfulness or metta interventions for PTSD and related comorbid disorders, including depression, anxiety disorders, or dissociative disorders.

In an effort to increase the availability of mindfulness- and metta-based interventions to persons with trauma and stressor-related disorders, we therefore developed a self-help internet resource we titled mindfulness and metta-based trauma therapy (MMTT). MMTT is intentioned primarily as a set of self-regulatory practices aimed toward augmenting outcomes associated with existing evidence-based treatments. The present report describes our initial development and “proof-of-concept” evaluation of the MMTT internet resource in the form of assessing participants’ immediate experiences in response to completing the therapeutic exercises contained therein, that is, upon their first visit to the website. Participants were recruited via the internet and varied with respect to the presence or absence of diagnosed history of psychological disorders and self-reported PTSD. A more detailed description of the MMTT website content is given within the methods section. Our research aims were to evaluate the perceived credibility of the website content and interventions, broadly for improving self-regulation and well-being, as well as more specifically for treating PTSD and related comorbid symptoms of anxiety, depression, and dissociation.

## Method

### Participants

Participants were recruited via Amazon’s Mechanical Turk (MTurk) web service which has been validated as a recruitment methodology for clinical psychology research (e.g., Shapiro et al. 2013). Importantly, all data collection, however, occurred on an external MMTT website such that participants’ responses were de-identified from their MTurk username credentials, maintaining the anonymity of data collection. Three hundred and two participants logged into the MMTT website, with 177 (59 %) meeting inclusion criteria for analysis via their completing the PCL-5 and at least one exercise. Participants were compensated nominally for taking part in the study via the MTurk website, thus maintaining confidentiality and data anonymity. Demographic details of the study sample are reported in Table 1.

**Table 1:** Demographic and clinical characteristics of the study sample

Sex:	
Female	118 (67.1 %)
Male	56 (31.8 %)
“Choose not to respond”	2 (1.1 %)
Age:	*M* = 35.30, SD = 11.30 (range 18–75)
Ethnicity:	
Caucasian (European)	128 (72.7 %)
“Mixed race”	16 (9.1 %)
“Other race”	10 (5.7 %)
Another specific race	<10
“Choose not to respond”	6 (3.4 %)
Employment:	
Employed part time or full time	93 (52.8 %)
Student	11 (6.3 %)
“Other”	69 (39.2 %)
“Choose not to respond”	3 (1.7 %)
Marital status:	
Single	74 (42.0 %)
Married/common law	72 (40.9 %)
Separated/divorced	19 (10.8 %)
Widowed	1 (0.6 %)
“Other”	7 (4.0 %)
“Choose not to respond”	3 (1.7 %)
Education:	
Fully completed post-secondary	94 (53.4 %)
Partially completed post-secondary	57 (32.4 %)
Secondary school only	22 (12.5 %)
Did not complete secondary school	0 (0 %)
“Choose not to respond”	3 (1.7 %)
Any psychiatric diagnosis:	
Never diagnosed	92 (52.3 %)
Past but not current	25 (14.2 %)
Current	51 (29.0 %)
“Choose not to respond”	8 (4.5 %)

### Procedure

All study procedures were approved by the Western University Health Sciences Research Ethics Board. Participants logged into the MTurk website and were presented with a link to the participation registration page of the MMTT website where they created a personal username and password before logging in (homepage presently at mmtt.ca). Upon logging in, participants were presented with an informed consent form describing the research objectives, procedures, data recording, limits to confidentiality, and potential risks of participating in the study, the latter conveyed as follows: “There is a risk that you will become uncomfortable and/or be distressed by the website content. If you are in duress, we recommend that you contact your local mental health crisis line or hospital emergency department.” Participants were also given the contact information of the study principal investigator should they wish further clarification regarding the informed consent process. After providing informed consent (via button press), participants were administered a simple demographic survey and also asked to complete the PTSD Checklist for DSM-5 (PCL-5; described below) in order to assess DSM-5 PTSD symptoms (results in Table 1).

Participants were then presented with video-recorded instructions on how to navigate the MMTT website and were asked to complete at least one exercise from each of four tabbed categories (described below). Participants were then free to choose whichever therapeutic exercises appealed to them from within the four tabbed categories (further described below): (1) journal, (2) MBAS meditation timer, (3) guided meditations, and (4) psychoeducation/instructions. It was suggested that, should they find any of the exercises distressing or unhelpful, they were free to discontinue completion of that exercise. Participants were presented with an “*Exercise Feedback Questionnaire*” after completing each exercise (described below). Study participation was considered completed when the participant freely logged out of the website. Although completion of the study procedure required only a single visit to the MMTT website, participants were informed that they would have unlimited, continuing access to the website should they wish to use it again for a period of 6 months following the study; subsequent log-ins, however, were not monitored for the purpose of this research project.

### Measures

#### MMTT Website

The MMTT website we evaluated herein (homepage presently at mmtt.ca) included four primary groups of interventions organized in a tabular structure: (1) journal, (2) MBAS meditation timer, (3) guided meditations, and (4) psychoeducation/instructions. The design and all website content was developed, written, and narrated for video and audio presentation by the first author. Programming was accomplished by the third author under the direction of the first author.

The journaling exercise was generally based upon the concept of an *automatic thought record* as used in cognitive therapy for mood and anxiety disorders (Beck 1976; Beck et al. 1979; Greenberger and Padesky 1995) but was explicitly adapted so as to facilitate the application of six therapeutic principles associated with mindfulness and metta therapies toward the adaptive reappraisal of distressing life events: (1) presence, (2) awareness, (3) letting go, (4) metta, (5) re-centering and de-centering, and (6) acceptance and change. The definitions of the six MMTT principles as provided to users of the website are included in Table 2. Participants were instructed that, via the MMTT journal, they could “record what happened during difficult or distressing life events or relationships and learn to reappraise their response to these events using the six MMTT principles.” It was further suggested that they might “find it helpful to journal about positive experiences as well.” Participants were first instructed to describe a personal life event in objective terms, given the suggestion to “include only the facts.” They then proceeded to describe their subjective experience in response to the event “in terms of feelings, thoughts, behaviors, and bodily reactions.” Finally, participants applied as many of the six MMTT principles as they determined were relevant in order to appraise their response to the event adaptively. Participants were provided video instructions on how they might do so, and two example journal entries were also provided for instructional aid, the latter also included in Table 2 for illustrative purposes. Upon clicking a “submit” button, participants’ journal entries were saved and, should they wish, could be viewed again later.

**Table 2:** Six therapeutic principles of MMTT and examples given in Journal exercise

		Example 1	Example 2
		Objective event: “Being late for an important appointment.”	Objective event: “Failed to completely stop at a STOP sign and was pulled over by a police officer.”
Principle	Definition	Subjective experience: “Feeling embarrassed, guilty, anxious. Thinking that: ‘I can’t do anything right’ and worrying that: ‘I’ll be yelled at’.”	Subjective experience: “Heart racing, panic, anger. Think to myself: “You idiot!!” Worry that: “The police officer will hurt me.”
1. Presence (vs. past)	Reminding yourself that you are in the present and recognizing the influence of the past on your way of relating to the present (i.e., recognizing the influence of past experiences, memories, and emotions on your responses in the here and now).	“Remembering that I am in the present – not in the past. The person or people at the meeting are NOT likely to yell at me or hurt me.”	“Remember that this is the present – that I am safe. Realize that, although I might get a ticket, it is NOT likely that I will be physically hurt.”
2. Awareness	Being experientially aware, especially of stimuli through your five senses and of your emotional states, primarily as a means of grounding yourself and being better able to label and understand your experiences and describe them to others.	“I can practice being aware of my feelings. I can stay grounded by flexing my fingers and toes.”	“Practice labeling my emotions: this is fear, this is anger. Be aware that I can feel my hands on the steering wheel of my car.”
3. Letting go	Allowing distressing experiences to pass by and recognizing and becoming less attached to acting upon harmful impulses or desires.	“I can practice letting go of my need to be perfect in others’ eyes. I can realize that everyone is late sometimes.”	“Practice letting go of my fear, anger, and self-criticism.”
4. Metta	Experiencing lovingkindness and compassion for yourself and trusted others.	“I can try to be kind with myself in this moment. I can realize that there is NO need for me to be embarrassed or ashamed.”	“Remember that everyone makes mistakes. Try to be compassionate to myself in this moment. And if I begin to feel angry toward the police officer, remember that he or she is only doing their job.”
5. Re-centering and de-centering	Feeling safe and at ease within your body, being able to feel a greater sense of pleasure and joy in your body, and being able to tolerate bodily feelings of distress.	“I can practice decentering from my feelings of guilt and embarrassment – I can realize that these feelings are NOT helping me right now.”	“Using decentering try to recognize that being self-critical or blaming will not help the situation.”
6. Acceptance and change	Recognizing the difference between things that cannot be changed and things that can and beginning to act upon things that can be changed.	“I can accept that I’m running late. I can plan to apologize and explain why I’m late when I meet the other person or people.”	“Accept that I will have to speak with the officer - Practicing change I can begin to plan what I will say, and try to be a more careful driver in the future…”

The second tabbed exercise category included on the MMTT website was a meditation timer designed to administer silent or audio-accompanied open or closed-eyes meditations of a participant-specified duration, as well as to measure *Meditation Breath Attention Scores* (MBAS; Frewen et al. 2008, 2011, 2014). MBAS represents a procedure by which a person’s ability to maintain their concentration during meditation practice can be assessed, that is, as directed toward an intended object (e.g., breathing) and away from distractions (e.g., mind wandering). Specifically, meditation bells were rung during the meditation practice and participants were instructed: “When you hear a meditation bell, click or touch anywhere on the screen if your attention was focused at that moment. However, if the bell alerts you to the fact that your attention has become distracted, simply do NOT click or touch the screen. Instead, label to yourself the source of the distraction and then practice letting go, refocusing your attention.” If a participant clicked/touched on their computer, tablet, or smartphone screen within 3 s following a meditation bell, they received an MBAS of 1 for that bell; otherwise, they received an MBAS of 0. The MMTT website acknowledged the recording of participants’ responses by resounding the bell, and upon completion of the meditation practice, participants could view the sum MBAS they received as a proportion of the number of meditation bells sounded during the meditation practice, not only for the meditation just completed but also for all previous uses of the meditation timer to afford comparison. It was suggested to participants that: “You may find that practicing in this way, over time, improves your level of meditative concentration, making your mind more focused and less prone to wandering and distraction.” Participants choose the duration of their meditation practice in minutes, the number of bell sounds that would be sounded, whether the bells would be sounded at equal or random intervals, the particular sound of the meditation bell that would be sounded (from a listing of .mp3 recordings of various Tibetan meditation bowls), what visual stimulus they preferred to fixate upon in case they wished to practice the meditation with their eyes open (from a list of .gif images, e.g., candle, mandala, nature scene), and whether they wished to include background audio during their meditation practice (from a listing of .mp3 recordings of natural sounds, e.g., rainfall, running stream, or brook).

The third tabbed exercise category included on the MMTT website was a listing of 13 guided meditations that could be played via a within-site video player, all between 8 and 10 min in duration. The meditations were loosely divided into those focusing primarily on the development of mindfulness and concentration, metta (lovingkindness), visualization and embodied imagery (termed *embodiment meditation*), and body awareness (i.e., *body scan*). Table 3 provides a brief description of the general content of each of the guided meditations. Finally, the fourth tabbed exercise category included a number of psychoeducational videos that (1) introduced a rationale for practicing mindfulness and metta as interventions for trauma and stressor-related disorders; (2) defined and applied the aforementioned six therapeutic principles to self-regulation; or (3) gave instructions concerning how other exercises available within the website could be completed.

**Table 3:** Guided meditations

Meditation type	Title	Brief description
Mindfulness, concentration	Breath counting	Participant guided in maintaining mindful attention toward both breathing and tactile awareness (touching fingers to thumbs) for 20 breaths.
Mindfulness	Mindful awareness of sight and sound	Participant guided in identifying, labeling, focusing upon, and finally letting go of their focus upon a single visual and a single auditory stimulus from within his or her immediate environment.
Metta	Freedom from pain and suffering	Lovingkindness meditation (LKM) with the following four intentions: (1) safety from harm, (2) freedom from suffering, (3) lessening of pain, (4) finding of peace.
Metta	Hope, joy, love within, peace within	LKM with the following four intentions: (1) having hope, (2) experiencing joy, (3) finding love within, (4) finding peace within.
Metta	Empowerment, strength, security, resilience	LKM with the following four intentions: (1) being empowered, (2) being strong, (3) being secure, (4) being resilient.
Embodiment	Mountain imagery	Participant first guided in visualizing and then physically embodying (via postural change) the following four characteristics of a mountain: (1) strength, (2) resilience, (3) wide point of view, (4) solidity.
Embodiment	Tree imagery	As for mountain imagery but with the following four characteristics of a tree: (1) expansiveness, (2) firmly rooted, (3) place of comfort, (4) natural beauty.
Embodiment	Sun imagery	As for mountain imagery but with the following four characteristics of the sun: (1) source of light, (2) pleasant warmth, (3) energy, (4) potential.
Embodiment	Holding a candle imagery	Participant guided in walking meditation while imagining themselves holding a lit candle symbolizing a part of themselves they may nurture and respect, embodying the following four characteristics of candles: (1) source of light, (2) pleasant warmth, (3) sign of comfort and calmness, (4) something that requires care.
Embodiment	Body scans	Participants guided in becoming aware of, labeling, and finally letting go of (i.e., releasing attention from) physical sensations in one of four focused areas of the body during four (separate) meditations: (1) lower body, (2) upper-middle body, (3) hands and arms, and (4) head and face. All body scan meditations also concluded with encouraging the experience of the entire body as a whole.

All guided meditations included repeated practice of breath-focused meditation. Participants were instructed that they may practice the meditations with their eyes open or closed, and either seated, lying, or standing, as they prefer.

*LKM* lovingkindness meditation.

##### Exercise Feedback Questionnaire

A 10-item 5-point Likert rating scale was administered immediately after participants completed each website exercise in order to assess participants’ experiences with each exercise. The item contents were modeled after surveys used in similar studies (Klein et al. 2009; Niles et al. 2012), satisfaction scales used in other research investigating internet therapy (Ruggiero et al. 2006), the *Credibility/Expectancy Scale* (Devilly and Borkovec 2000), and the *Treatment Satisfaction Scale* (Cox et al. 1994). Specifically, participants indicated their level of agreement (from “Disagree” to “Strongly Agree”) regarding whether the website exercises were (1) “distressing (e.g., made me anxious or upset),” (2) “credible as a way to improve my self-regulation and enhance my well-being,” (3) “credible as an intervention for anxiety, depression, PTSD, and dissociation,” (4) “easy to complete,” (5) “helpful,” (6) “informative,” (7) “calming,” and (8) “enjoyable”. Two additional questions inquired whether the participant would “recommend this exercise to a friend” and “complete this exercise again”. Text boxes for collecting open comments were also presented with participants asked to indicate what they may have learned and experienced while completing each exercise, as well as a field for general comments. For the present study, due to the fact that the exercise feedback questionnaire was to be repeatedly administered, that is, upon completion of each specific website activity that participants completed (i.e., completion of each journal entry, MBAS meditation timer meditation, guided meditation, and information video), it was felt that a brief measure of direct pertinence to the website content was required.

##### PTSD Checklist for DSM-5 with TRASC Item-Addendum

The 20-item PTSD Checklist for DSM-5 (PCL-5; Weathers et al. 2013) was administered. The PCL-5 is a measure of self-reported DSM-5 PTSD symptoms, with responses given on a frequency scale between “*Not at all*” (scored 0) and “*Extremely*”(scored 4). The DSM-5 PTSD diagnosis includes 20 symptoms, hence the 20 PCL-5 items (i.e., the PCL-5 has a symptom to test-item ratio of 1:1). Total scores on the PCL-5 were calculated as the sum of scores for all 20 items, with a score of 38 recommended as a cut-off for probable PTSD (Hoge et al. 2014; Weathers et al. 2013). The internal consistency reliability observed in the present study was excellent (*α* = 0.97).

### Data Analysis

To simplify analyses of ratings in response to completion of the guided meditations, responses to the three metta meditations, four embodiment meditations, and four body scan meditations were averaged (see Table 3). Response to the website exercises were then compared in terms of mean *Exercise Feedback Questionnaire* ratings between groups of varying self-reported PTSD symptoms (*PCL-5* scores) via between-subjects ANOVA, specifically, between participants with low/absent (PCL-5 ≤ 14), mild/moderate (PCL-5 15–37), and high (PCL-5 ≥ 38) PTSD symptom severity. The significance of correlations between PTSD symptom severity and feedback ratings was also evaluated. In addition, response to different exercises was compared within each of the three PTSD symptom severity groups via paired *t* tests, specifically, by comparing ratings referring to each individual exercise type with the mean of the seven other categories. To reduce multiple comparisons and associated risk of type-1 error, the 10-items of the *Exercise Feedback Questionnaire* were averaged, with reverse scoring of the first item pertaining to experiences of distress. Supporting such averaging, internal consistency reliabilities were high when calculated in response to all exercise types (*α* > 0.80). All statistical analyses were 2-tailed.

## Results

In total, data were collected from 640 exercises. On average, participants completed 3.89 exercises (SD = 1.09, range 1 [*n* = 9] to 10 [*n* = 1]); most participants completed either three (*n =* 91, 51 %) or four (*n =* 46, 26 %) exercises. PCL-5 scores ranged from 0 to 76 (*M* = 21.37, SD = 19.22), with 86 participants (49 %) scoring in the non-PTSD range (PCL-5 ≤ 14), 53 (30 %) scoring in a mild-to-moderate PTSD symptom range (i.e., PCL-5 score between 15 and 37), and 37 participants (21 %) scoring at or above the cut-off score of 38 recommended for studies of the population prevalence of PTSD (Hoge et al. 2014; Weathers et al. 2013).

**Fig. 1:**
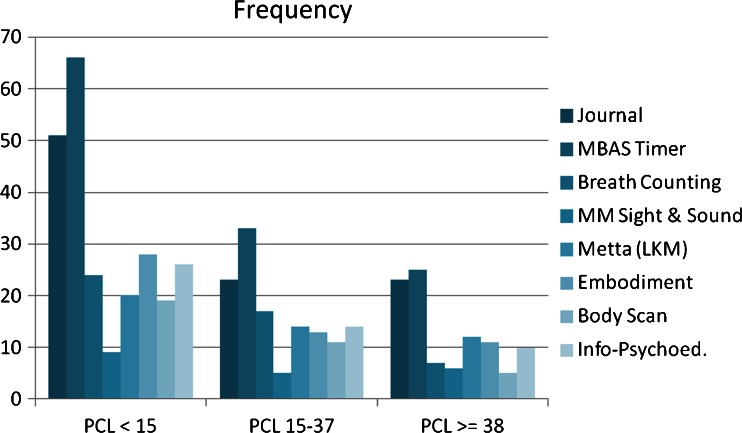
Number of participants who completed each of eight different MMTT exercises across three PTSD symptom severity groups

Figure 1 shows the number of exercises completed by participants with low/absent (PCL-5 ≤ 14), mild/moderate (PCL-5 15–37), and high (PCL-5 ≥ 38) PTSD symptom severity as referring to the eight exercise categories. A majority of participants completed the journaling exercise, the MBAS meditation timer, and at least one form of guided meditation. However, only a minority provided feedback regarding the informational-psychoeducational videos.

**Fig. 2:**
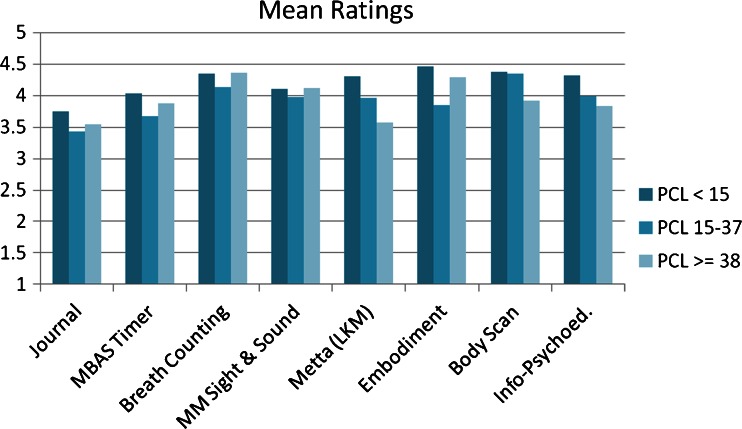
Mean feedback survey score for each of eight different MMTT exercises across three PTSD symptom severity groups

Representative open-ended responses to the question “What did you learn or experience while completing this exercise?” in reference to each of the eight exercise categories are included in Table 4 separately for the three PTSD symptom severity groups. Figure 2 shows the corresponding results for the 10-item *Exercise Feedback Questionnaire* ratings. One-way ANOVAs indicated no significant differences between groups for ratings for any exercise type, although there were non-significant trends (*p <* 0.10) for group differences in ratings referring to metta (lovingkindness) meditations, *F*(2,43) = 1.95, *p* = 0.067, and embodiment meditations, *F*(2,49) = 1.68, *p* = 0.068; all post hoc tests in these cases, however, were also non-significant. Correlations between PCL-5 scores and *Exercise Feedback Questionnaire* ratings referring to each of the eight exercise categories, however, showed a negative correlation between total PTSD symptom severity and positive feedback ratings for metta meditations, Spearman’s *rho* = −0.39, *p* = 0.007 (2-tailed); all other correlations were non-significant (*p’*s ≥ 0.09).

**Table 4:** Feedback survey results (qualitative, open-ended ratings)

Exercise	Group	Example response to: “What did you learn or experience while completing this exercise?”
1. Journal	LOW	How to work through my feelings and visualize what I need to do to make changes.
		This opened up a lot of past wounds. I guess this helped me clarify some feelings but it more felt like reliving bad feelings that I try not to think about too much. Perhaps, doing this exercise multiple times would bring clarity.
	MED	I learned that I am nervous and tense, which I did not notice before. Writing about how I feel makes me pay more attention to that.
		I experienced how to become fully aware of all the anxious and complex feelings I have been having. It really helped to write it out, to understand, and helped me to learn how I can rid myself of these particular fears and that everything really is going to be ok. Also, how I can let go of the past, accept what is, and how I will strive to change for the better and love myself still no matter what. I also taught myself that remaining calm and not panicking will definitely help because I waste time in states of panic and anxiety instead of actually focusing and getting work done. This really did help some to get all this out of me in words.
	HIGH	Although I remembered a past experience vividly and knew it bothered me, I had never connected it to my difficulty making phone calls before. I learned of the connection, and for the first time, how to talk to myself to move past it. It didn’t make the problem go away, although over time I believe that this kind of attention and thinking through it will help significantly.
		That I had a lot more to say than I ever would have thought. I usually have an extremely difficult time expressing myself in words…likely because I go for weeks without really saying anything. Putting emotions and thoughts into words is hard, but I managed to just let my stream of consciousness take over. Perhaps I should do this more often.
2. Meditation Timer (MBAS)	LOW	I experienced an empty mind that was focused on a singular image, which allowed my head to clear.
		I became very aware of myself, feeling each breath.
	MED	I learned how to completely focus on one assigned object while forgetting most of what was going on around me and/or anything on my mind.
		It was a new experience. I’ve never tried meditation before. I think it helped me calm myself and take negative thoughts from my head and helped me let go of them.
	HIGH	The experience helped clear my mind. I started with thoughts and then I let go and focused on the candle. (Freed my mind in some sense)
		My mind never stopped chattering. It was chattering about the exercise so I guess that’s better than totally drifting away to something else. I know this will take some time and practice.
3. Breath Meditation	LOW	The repetition of touching my fingers and breathing was very relaxing, and I feel like I have more clarity. It’s kind of difficult to express, so that’s the best way I could think of to describe the feeling.
		It was a very relaxing thing to do—it just made me focus on the activity and made me clear my head from anything distracting or unnatural.
	MED	That focusing on how I held my hands during the meditation helped me focus my mind. I also noticed that I don’t breathe deeply and worked to correct this during the meditation.
		To be aware of the nerves in my fingertips and thumb and to regulate my breathing.
	HIGH	It helped me block out things and focus.
		To clear my thoughts, let go of anything that is bothering me. Enjoy the calmness of quiet and relaxation.
4. Mindful Seeing & Hearing	LOW	I experienced peace. I felt very comfortable and really noticed sounds around me that I do not usually notice.
		I noticed how comfortable the sounds of my room can be if I maintain a calm mind.
	MED	How to attend to my senses.
		I felt like I was experiencing only one sense at a time which was pretty cool.
	HIGH	I learned about recognizing sounds and sights around me.
		How to quiet voices in my head. How to breathe effectively.
5. Metta Meditation	LOW	I felt lots of positivity going through me, and I especially loved when I “sent” hope and love and joy to someone.
		How to focus more on my thoughts (wishes, requests) for healing.
	MED	How to reinforce that I am safe if I am overwhelmed—the four affirmations refocuses your thought from panic to calm.
		Empowerment, felt more grounded and secure, very relaxed, no intrusive thoughts.
	HIGH	I learned how to accept positive affirmations for myself and others.
		It was difficult for me to do this practice. I had a difficult time taking in metta than sending it out to my mother. I had a hard time relaxing.
6. Embodiment Meditation	LOW	By imaging something representative of positive traits and putting yourself in a position to feel that way, you can gain positive energy and be in a better position to feel more confident.
		To be fully present, feeling safe and strong, encompassing the beauty and strength of a tree. For me, I learned that I can use this imagery for myself when faced with future encounters with my sister.
	MED	I learned that I am my own foundation. Standing tall like a mountain. Mountains are often a symbol of strength, and I imagined myself as having some qualities of a mountain. I tried to contact my inner strength by withstanding the troubles in my own life.
		I experienced a lot of imagery. I realize that if I close my eyes, the images I make in my mind from only hearing the guided meditation are an expression of my seemingly limitless creativity.
	HIGH	I loved how this meditation brought the strength of a mountain to myself.
		I could actually feel warmth surrounding me and moving through me. It felt really good. I’ve learned that the feelings of darkness within me aren’t permanent. It is possible for me to consciously change my thoughts and my mood.
7. Body Scan	LOW	It made me more aware of my body as a whole. Usually when I am stressed I think it is all mental, this made me realize I carry a lot of tension in other areas of my body as well.
		I really focused on the lower part of my body. Even though I didn’t even touch my legs, I feel very relaxed—as though I had a massage.
	MED	I had a peaceful and calm feeling come over the different parts of my body.
		I felt more in control of my body and its experiences. My hands have never felt so relaxed!
	HIGH	I was more in tune with my body. I could feel different sensations throughout.
		That taking a moment to focus on my body, I can relax the different muscle groups, making me more relaxed, calm, and peaceful.
8. Info. / Psychoed.	LOW	Learning how to use the techniques such as guided imagery or journal writing, to heal from and move on from a life altering event such as post-traumatic stress, bereavement, being verbally or physically abused.
		I learned about a case study—“Marie”. Though she had experienced very traumatic events, and had PTSD, she seemed to be able to use the techniques to reduce her pain.
	MED	Through this, I learned that self-help for traumatic life events or relationships is beneficial for my health and well-being.
		I found it interesting to follow along with Marie’s progress [a case study] and see how her beliefs and way of thinking began to change. I am glad that she was able to find some peace and that her PTSD symptoms were greatly diminished.
	HIGH	These are techniques that I feel need to be practiced, and the more you do the more you will benefit. They almost seem to bring your mind, body, and soul all together. My recent therapist does not utilize these, but I would like to start.
		Wow, that was really powerful [case study of “Marie”]. I cried but it wasn’t necessarily a bad thing. Empathy can be good. I learned that over time these exercises can lead to real recovery.

*Exercise Feedback Questionnaire* ratings were also compared between different exercise types separately within each of the PTSD symptom severity groups. Results showed that the journaling exercise was preferred less in comparison with other exercises within the low/absent, *t*(48) = 3.87, *p* < 0.001, and mild/moderate PTSD symptom groups, *t*(20) = 2.74, *p* = 0.013, with a similar (although non-significant) trend also observed for the high PTSD symptom severity group, *t*(20) = 1.96, *p* = 0.064. In comparison, the *breath counting* meditation was rated more positively than the average of all other exercises by all three groups: low/absent symptoms, *t*(23) = 2.24, *p* = 0.035, mild/moderate symptoms, *t*(16) = 2.69, *p* = 0.016, and high symptoms, *t*(6) = 3.20, *p* = 0.019. Body scan meditations were rated above average by the mild/moderate symptom severity group, *t*(10) = 2.89, *p* = 0.016, with a similar (though non-significant) trend observed for the low/absent symptom group, *t*(18) = 2.06, *p* = 0.055. Finally, the low/absent symptom group rated embodiment meditations above average, *t*(27) = 2.59, *p* = 0.015; no other comparisons were statistically significant.

## Discussion

The present study developed and provided a “proof-of-concept” evaluation of mindfulness and metta-based trauma therapy (MMTT) in the form of an internet resource intended to support meditation practices relevant to the treatment of trauma and stressor-related disorders including PTSD and related comorbid disorders such as depression, anxiety disorders, and dissociation, as well as more generally for improving self-regulation and psychological well-being. We assessed participants’ immediate experiences of the MMTT exercises during their first visit to the website.

In general, the results of this pilot study suggest that participants considered the MMTT website as a highly credible and satisfactory self-help resource for meditation practice as well as for the learning and application of mindfulness and metta-related therapeutic principles to daily life. Participants tended to rate the website exercises as highly relevant, calming, enjoyable, and informative. With few exceptions, similar ratings were found irrespective of participants’ self-reported PTSD symptom severity, suggesting that the website exercises were considered potentially helpful not only in the context of treatment for trauma and stressor-related disorders but also more broadly in support of persons seeking to improve their self-regulation and well-being within the general population.

The aforementioned positive findings notwithstanding, at least two caveats are pertinent to note. Firstly, we found that participants with more severe PTSD symptoms tended to rate the practice of metta (lovingkindness) meditations somewhat less favorably. Given that the practice of metta meditations involved rehearsing positive affirmations directed toward both self and trusted others, such practices may have been distressing to those who felt unworthy of such intentions due to trauma-related guilt or shame, such as may develop through intentional trauma, abuse, and neglect (e.g., Frewen and Lanius 2015). For example, research has shown that traumatized persons often respond to signs of others’ affection or good intentions with distress and negative affect, rather than with expected positive affect, for example, upon receiving a gift or being thanked or praised for a good deed (Depierro et al. 2014; Frewen, Dean, and Lanius, 2012; Frewen, Dozois, and Lanius, 2012). Although practice of metta meditations may initially engender distress in certain traumatized persons, with time they may come to be more accepting of the positive affirmations. Indeed, Kearney et al. (2013) found that a 12-week course in metta meditation as an adjunct to treatment as usual improved PTSD symptom reduction and increased self-compassion and mindfulness traits in combat veterans with PTSD. Further study of the therapeutic potential of metta meditations and therapies for persons with PTSD is needed.

A second qualification regards our findings that certain website exercises were rated more favorably than others overall. For example, a breath counting meditation in which participants were guided in maintaining mindful attention toward both breathing and tactile awareness (the latter via touching their fingers to their thumbs) was rated above average independent of PTSD symptom severity. Additionally, particularly among persons reporting lower or absent PTSD symptoms, body scan and embodiment meditations were also rated favorably relative to the average of other exercises. Comparison of the perceived benefits of different types of meditation practice is an area open for future research.

In contrast, overall, participants tended to provide lower feedback ratings for the journaling exercise in comparison with other website content. This result is interesting in light of prior research showing that completion of automatic thought records are generally rated as a less preferred therapeutic activity relative to other interventions within the context of CBT for affective disorders (e.g., in comparison with behavioral experiments/activation; Bennett-Levy 2003; McManus et al. 2012), and some researchers’ reasoned doubts regarding the unique value and necessity of inclusion of the more explicitly “cognitive” interventions within CBT more generally (e.g., Longmore and Worrell 2007). However, on balance, several participants described clearly benefitting from completion of the journaling exercise with regard to their reflection and newfound insight upon a matter of importance to them. Indeed, we investigated the kinds of life events participants chose to apply the journaling activity toward and found that whereas many chose to journal only about everyday and relatively neutral events, others chose to apply the journaling exercise toward potentially highly traumatic life events and stressors, for example, as drawn directly from responses to the “objective events” field: “marital infidelity,” “death of mother/father,” “spending time in jail,” “being sexually assaulted,” and “childhood molestation for 3 years”. It is further important to note that journaling about apparently more serious life stressors was *not* associated with higher distress ratings or appreciably lower overall feedback ratings in comparison with journaling about comparably neutral life events, suggesting that the exercise might be directly applicable to traumatic memory focused therapy. Given that the current structure of the MMTT website provides for the flexible use or non-use of different therapeutic exercises entirely in accordance with participant preference, we expect to retain the journaling exercise in future evaluations of the website.

There are obvious limitations to what can be concluded about the value of the MMTT website based on this initial proof-of-concept project alone. For example, our feedback questionnaire mostly included positively framed items and an acquiescing response would therefore favor a positive response bias; future studies would be well advised to utilize a previously validated questionnaire (e.g., Devilly and Borkovec 2000). In addition, researchers may wish to evaluate whether different kinds of exercises (e.g., meditation practice vs. journaling vs. psychoeducation) are associated with different kinds of therapeutic outcomes (e.g., informative vs. experiential). In addition, although there were no reports of significant distress engendered through participation in the website exercises included in this study, it would not be possible, due to the anonymity of the data collection procedure, for clinician researchers to directly intervene should such distress occur; adequate crisis planning as such should be in place when participants are completing online psychological interventions. Limitations of this study further include: that we only monitored participants’ first use of the website rather than repeated visits over time; that all data was acquired via self-report and may be biased due to demand effects or otherwise; that no control or comparison website was evaluated as a means of identifying whether any benefits of the MMTT website are unique; and that sample sizes were small particularly in reference to persons reporting more severe PTSD symptoms. The results of the present study should therefore be considered primarily as a means of hypothesis generation for future research and replication. Stronger evidence would be provided by larger randomized trials evaluating response to the MMTT website over time and across repeated sessions, in comparison with a control intervention. Such studies should evaluate PTSD outcomes assessed not only by self-report but also by clinician rating and in comparison with or as an adjunct to traditional psychotherapy. Moreover, evaluation of response to the website in persons with clinically significant PTSD is needed, given that, within the present study, participants were relatively homogenous with regard to demographic characteristics and only a minority reported high symptom severity.

In clinical settings, different uses of the website can be envisioned, for example, as a more or less clinically integrated adjunctive intervention to support evidence-based in-person psychological treatment, or as a means of partly structuring the delivery of in-person individual or group mindfulness- and metta-based therapy. Moreover, the website content and design was developed for transdiagnostic application, that is, in the hope of being of potential therapeutic benefit to persons with various mental health needs, as opposed to being an intervention of relevance only to one or more specific psychological conditions. However, to be clear, it is the authors’ clinical opinion that therapeutic resolution of clinically significant post-traumatic experiences usually requires reflection on how past traumatic events are affecting an individual’s capacity to function in the present, as well as some form of working through of the traumatic event within the context of a therapeutic relationship with a skilled and compassionate clinician. It is our further assumption that this will usually transpire most humanely and effectively through the naturally human mode of face-to-face communication and empathic support. Generally, consistent with this, a meta-analysis by Sloan et al. (2011) of studies comparing PTSD outcomes following telehealth therapy with traditional face-to-face treatment found that although telehealth interventions were significantly more effective than wait-list control, they were *not* associated with significantly better outcomes when compared with non-specific supportive counseling and were associated with significantly *poorer* outcomes when compared with evidence-based therapies delivered face to face (see also Morland et al. 2014, for review). As such, we expect that PTSD treatments delivered via non-traditional methods, including the internet, rather than replacing in-person treatment, are likewise more appropriately applied adjunctively as a means of potentially augmenting and supporting ongoing face-to-face therapy, as well as preventing relapse following the conclusion of face-to-face treatment. Whether an added clinical benefit to persons with PTSD can be procured through alternative intervention modalities such as the internet, however, particularly as adjunctive psychological interventions, remains a highly important question to investigate.
